# Reduction in Virulence over Time in *Ostreid herpesvirus 1* (OsHV-1) Microvariants between 2011 and 2015 in Australia

**DOI:** 10.3390/v13050946

**Published:** 2021-05-20

**Authors:** Georgia Cain, Olivia Liu, Richard J. Whittington, Paul M. Hick

**Affiliations:** Sydney School of Veterinary Science, The University of Sydney, Camden 2570, Australia; gcai8763@uni.sydney.edu.au (G.C.); Olivia.Liu@awe.gov.au (O.L.); richard.whittington@sydney.edu.au (R.J.W.)

**Keywords:** Pacific oyster, *Crassostrea gigas*, *Ostreid herpesvirus 1*, virulence, phenotype

## Abstract

Microvariant genotypes of *Ostreid herpesvirus 1* (OsHV-1) are associated with mass mortality events of Pacific oysters in many countries. The OsHV-1 microvariant (µVar) emerged in France 2008 and caused significant economic losses as it became endemic and displaced the previously dominant OsHV-1 reference genotype. Recently, considerable genotypic variation has been described for OsHV-1 microvariants, however, less is known about variation in viral phenotype. This study used an in vivo laboratory infection model to assess differences in total cumulative mortality, peak viral load, transmissibility, and dose-response for three OsHV-1 isolates obtained between 2011 and 2015 from endemic waterways in Australia. This followed field observations of apparent reductions in the severity of mass mortalities over this time. Significantly higher hazard of death and cumulative mortality were observed for an isolate obtained in 2011 compared to isolates from 2014–2015. In keeping with other studies, the hazard of death was higher in oysters challenged by injection compared to challenge by cohabitation and the mortality was higher when the initial dose was 1 × 10^4^ OsHV-1 DNA copies per oyster injection compared to 1 × 10^2^ DNA copies. There was no difference in the quantity of OsHV-1 DNA at time of death that could be related to isolate or dose, suggesting similar pathogenetic processes in the individual oysters that succumbed to end-stage disease. While the isolates examined in this study were biased towards pathogenic types of OsHV-1, as they were collected during disease outbreaks, the variation in virulence that was observed, when combined with prior data on subclinical infections, suggests that surveillance for low virulence genotypes of OsHV-1 would be rewarding. This may lead to new approaches to disease management which utilize controlled exposure to attenuated strains of OsHV-1.

## 1. Introduction

*Ostreid herpesvirus 1* is a member of the family *Malacoherpesviridae* within the order *Herpesvirale* [1]. Microvariant genotypes of Ostreid herpesvirus-1 (OsHV-1) are of international concern because they cause more severe disease than the reference genotype and have spread rapidly [2,3]. Compared to the reference genome (GenBank accession number AY509253), the microvariants have been characterized by a deletion in the microsatellite locus upstream of open reading frame 4 (ORF 4) and several variations to the genome, including polymorphisms in ORF 4 (C region) and ORF 42/43 which encode an inhibitor of apoptosis [2]. Since the emergence of the microvariant genotypes in France in 2008 [4], OsHV-1 has spread between regions and across hemispheres with mass mortality outbreaks in Europe [5,6], New Zealand [7], and Australia [8,9]. In regions where the microvariant genotypes have been detected, they have become endemic and caused recurrent seasonal disease which is more severe than previous summer mortality events [10]. The microvariant genotypes have replaced the reference genotype, becoming the dominant cause of global mortality events in Pacific oysters [4,11]. The high mortality and seasonal reoccurrence of disease caused by OsHV-1 has led to significant economic losses in many countries and is driving the restructure of many Pacific oyster industries, which accounts for a large proportion of global edible oyster production [3].

In regions where OsHV-1 has become endemic, disease expression varies year-to-year and there can be significant differences in the level of mortality in different locations. Epidemiological studies have shown this is driven by the multifactorial nature of the disease, depending on risk factors relating to the environment and the host [12,13,14]. Importantly, not all OsHV-1 infections result in mortality [15,16]. This includes a growing appreciation of the polymicrobial pathogenesis of the disease subsequent to immune suppression due to OsHV-1 infection [17]. Several host factors contribute to the expression of OsHV-1, including the genotype [18,19] and the age and size of the oysters [20,21]. Abundant food is a risk factor for higher mortality in oysters as rapid growth and high metabolic demand can enhance disease, although disease resilience can also be promoted through building the oysters’ energetic reserves [22]. Food availability can also enhance OsHV-1 transmission [23]. 

Concerning the environment, water temperature is a key factor for disease expression with mortality events observed between 16–24 °C in Europe [24,25] and 22–25 °C in Australia [3,12]. Salinity also impacts OsHV-1 disease expression because the osmo-conformation of oysters can result in detrimental metabolic changes depending on the rate of change of salinity [26] and virus transmission by impacting the persistence of OsHV-1 infectivity [27]. Physical stresses, including handling during farming procedures and differences between water and air temperature, can increase OsHV-1 mortality [6,28]. Changes to farm management have been implemented to alter the host and environmental risk factors and minimize disease impacts. For example, raising the height of growing infrastructure decreased the immersion time for oysters in an intertidal environment, impacting growth, feeding, and exposure time for transmission of OsHV-1 [29].

Increased surveillance has identified considerable genotypic variation within the OsHV-1 species. Partial genomic sequences have revealed multiple genotypic variants within the microvariant and reference genome clusters [30], including considerable viral diversity in oysters without signs of disease [31]. While there has been less study of complete genomes, one microvariant genotype was shown to be 94.4% similar across 205 kilobases compared to the reference genome [32]. OsHV-1 genomic diversity is higher over greater geographical distance and more genotypic variation has been described in South Korea and China compared to Europe [33]. In China, the genome of Acute Viral Nervous Necrosis (AVNV), which causes severe mortality in adult Farrer’s scallops (*Chlamys farreri*) was shown to be a variant within the species OsHV-1 [34]. An additional variant was also identified in blood clams (*Scapharca broughtonii*) [35], illustrating the considerable scope for differences in OsHV-1 phenotype. However, the implications of this genotypic diversity on OsHV-1 disease outcomes requires further investigation. Phenotypic variation of OsHV-1 at a finer scale was suggested by differential mortality of Pacific oysters from a common source when they were infected with genotypically distinct OsHV-1 after placement in different locations in France [36]. Under experimental conditions, mortality of oyster families from well characterized breeding programs had different disease outcomes when exposed to a French OsHV-1 microvariant isolate compared to a non-microvariant OsHV-1 isolate from the United States, albeit under very different environmental conditions [37]. Similarly, differential mortality under experimental conditions was demonstrated for two different OsHV-1 microvariant strains originating from France and Australia [38]. 

**Figure 1:**
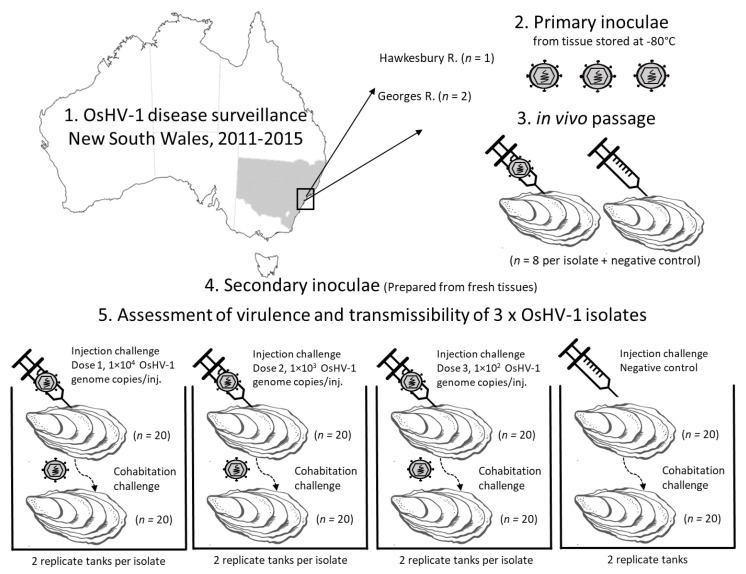
Experiment design to compare isolates of microvariant OsHV-1. Primary inoculae were prepared from oyster tissues collected during Pacific oyster mortality disease surveillance that were stored at −80 °C. Primary in vivo passage of each isolate by injection of Pacific oysters from the same hatchery and batch origin was used to reduce the impact of storage time, stage of infection, and status of primary host. The virulence and transmissibility of each OsHV-1 isolate was then compared using injection challenge with three doses of the secondary inoculae and by cohabitation with these injected oysters in replicate tanks.

The impact of OsHV-1 has been severe in two oyster producing waterways in New South Wales (NSW), Australia. The initial outbreaks in the Georges and Hawkesbury Rivers arose from probable single-point introductions of microvariant OsHV-1 and caused mortality exceeding 95%, with both farmed and wild Pacific oysters affected [8,9]. On-going surveillance in these estuaries revealed that OsHV-1 was endemic with recurrent seasonal outbreaks of disease [16]. The host population remained low due to reduced stocking of farmed Pacific oysters, generating selection pressures for OsHV-1 that may explain, in part, the reported diminution in mortality rates and prevalence of infection over the period 2012 to 2017 [16]. The present study was designed to evaluate whether attenuation of OsHV-1 could explain decreasing severity of disease in these endemically infected waterways. Isolates of OsHV-1 collected several years apart were evaluated for differences in phenotype using a standardized laboratory infection model for in vivo infection of Pacific oysters (Figure 1). Phenotypic differences between isolates were identified based on total cumulative mortality, transmissibility, incubation period, and peak viral load.

## 2. Materials and Methods

### 2.1. Oysters

Triploid juvenile Pacific oysters (six months of age; shell length 3–4 cm) were sourced from a commercial hatchery (Shellfish Culture, Tasmania, Batch SPL16A) and grown under commercial conditions by a farmer in Patonga Creek, NSW. Using the same batch of oysters grown under identical conditions ensured the genotype and previous environmental experience of the oysters was not a factor when comparing the disease outcome. These oysters had not been previously exposed to OsHV-1 based on: certification of freedom from OsHV-1 at the source hatchery by the government authority in Tasmania; no detections of mortality caused by OsHV-1 at the farm location during long-term surveillance [3]; and negative qPCR tests on a subsample of oysters when they entered the laboratory (*n* = 30).

### 2.2. Laboratory Housing 

Oysters were randomly distributed to eight tanks for the primary OsHV-1 amplification (*n* = 8) or to 26 tanks for the detailed comparison of OsHV-1 isolates (*n* = 40 per tank) and were acclimated to the physical containment level 2 aquatic animal facility for seven days. Each tank contained 15 L artificial seawater (ASW) at 30–31 ppt salinity (Red Sea salt) and was maintained separately with an individual airlift biofilter. The water temperature was maintained at 22 °C by immersing tanks in circulating water baths [39]. Oysters were fed twice daily with 2 ml/tank of Shellfish Diet 1800 (Reed Mariculture, Campbell, CA, USA). Water quality was monitored every second day and adjustments were performed as required by water exchange to maintain pH > 8.0 and ammonia, nitrite, and nitrate levels <0.25 ppm. Oysters within each tank were randomly assigned to either challenge by injection (*n* = 20) or challenge by cohabitation (*n* = 20), based on their position in the tanks. The injected oysters were identified by engraving a small notch on the upper shell. Treatment groups were randomly assigned to tanks.

### 2.3. Isolates of OsHV-1

Oysters infected with OsHV-1 obtained during previous research were selected to represent the greatest available range in time and geographical location of outbreaks (Table 1). The samples were obtained from disease outbreaks where Pacific oyster mortality syndrome was confirmed by the presence of microvariant OsHV-1 according to the OIE definition [2]. These oysters were stored as whole soft tissues at −80 °C. Samples stored as tissue homogenates without cryoprotectants were not included as OsHV-1 could not be amplified in vivo from these samples (data not shown). 

Secondary inoculae were prepared for the main experiment as fresh tissue homogenates from in vivo passage of the primary inoculae to minimize possible variation between the OsHV-1 stocks based on duration of storage, stage of infection, and the age/size of the original host.

#### 2.3.1. Primary Inoculae

The primary inoculae were freshly prepared, clarified, and filtered homogenates of mantle and gill tissue. Oysters were thawed at 4 °C, opened by removing the superior valve and the excised gill and mantle tissues were weighed and placed into a stomaching bag with 10× volume of sterile ASW. Tissues were homogenized in a stomaching machine (MiniMix, Interscience, France) at maximum speed for 1 min. The homogenate was then transferred into 50 mL sterile tubes and centrifuged at 1000× *g* for 10 min at 4 °C (Beckman Coulter, Brea, CA, USA). The supernatant was diluted with three volumes of ASW and filtered successively through 5 µm, 0.8 µm, 0.45 µm filters and twice through a 0.22 µm syringe filter (Sartorius, Göttingen, Germany). The clarified tissue homogenate was then stored briefly at 4 °C awaiting quantification of OsHV-1 DNA by real-time quantitative PCR (qPCR) prior to use. Tissues were processed in a class 2 biosafety cabinet with cross contamination between specimens and control groups prevented by use of autoclaved or sterile disposable equipment and surface disinfection using 1% Virkon-S for 15 min.

#### 2.3.2. Secondary Inoculae

The secondary inoculae were prepared by passage of the primary inoculum in vivo. Oysters that had been acclimated at 22 °C for three days (*n* = 8) were relaxed by immersion in 50 mg/L MgCl_2_, in 1 L of artificial sea water and 4 L of distilled water after being removed from water for 12 h. The primary inoculae were diluted with ASW to contain 10^4^ OsHV-1 DNA copies in 100 µL and were injected into the adductor muscle using a 1 mL syringe and 25-gauge needle. Twice daily examination identified dead oysters as those that were gaping and failed to close when removed from the water for 2 min; these were stored at −80 °C. Remaining oysters were collected when mortality reached 50% and were stored at −80 °C. Secondary inoculae were prepared from pools of gill and mantle from all of these oysters using the same method as the primary inoculae. OsHV-1 DNA in the resulting inoculums was quantified by qPCR (Table 1). The negative control inoculum was prepared from OsHV-1 free Pacific oysters using the same method and dilution factors described above.

### 2.4. Assessment of Virulence and Transmissibility of OsHV-1 Isolates

For each isolate there were two replicate tanks for each of three doses of OsHV-1. Twenty oysters per tank were infected by injection and 20 per tank were exposed through cohabitation. Sample size was designed to identify a 20% difference in mortality with 80% power and 95% confidence (https://epitools.ausvet.com.au/twoproportions (accessed on 18 May 2019). Oysters for injection were relaxed with MgCl_2_ and injected with 100 µL of the secondary inoculum diluted to contain 1.0 × 10^4^, 1.0 × 10^3^, or 1.0 × 10^2^ OsHV-1 genome copies per injection. Oysters were assessed twice daily by visual inspection and dead oysters were removed and stored at −80 °C. All oysters that survived to 14 days post injection were also stored at −80 °C until qPCR analysis. 

A previously described qPCR was used to determine the concentration of OsHV-1 DNA in each inoculae. The first five oysters to die in each replicate tank for each challenge method and a selection of five oysters from each replicate tank that survived until the end of the trial were tested to evaluate the quantity of OsHV-1 in each treatment group. 

### 2.5. Detection and Quantification of OsHV-1 DNA

Quantification of OsHV-1 DNA was according to previously described methods based on homogenization of equal parts of gill and mantle tissue (0.08–0.12 g) by bead beating and purification of nucleic acids using the MagMAX^TM^-96 Viral RNA Isolation Kit (ThermoFisher Scientific, Waltham, MA, USA) and a MagMax Express-96 magnetic particle processor [23]. The qPCR assay described by Martenot et al. [40] was used as previously described with duplicate 25 µL reactions prepared using the Path-ID qPCR kit (ThermoFisher Scientific) and thermocycling with an Mx3000 P Real-time PCR machine (Stratagene, La Jolla, CA, USA) [23]. Samples were considered positive for OsHV-1 when the ROX normalized and the baseline corrected FAM signal increased exponentially above the threshold and the quantity in positive samples was determined using a quantitative curve for the standard (plasmid pOSHV1-Breg). 

### 2.6. Statistical Analysis 

Total cumulative mortality between tanks was compared by ANOVA. The time of death for cohabitation was adjusted by −60 h from the time of injection to approximate the time of exposure to OsHV-1. Kaplan Meyer survival curves were prepared for each isolate, dose, and challenge method in R Studio using the ‘survfit’ function in the ‘survival’ package (https://cran.r-project.org/web/packages/survival/index.html (accessed on 5 October 2019)). Survival curves were assessed for significance using a log rank test (*p* < 0.05). A Cox proportional hazards model was prepared considering the factors isolate, challenge method, and dose, with tank as a random effect using the ‘coxph’ function in the ‘survival’ package for R [41]. The quantities of OsHV-1 DNA measured by qPCR were log10 transformed to meet the assumption of normality. Box plots with the median and quartiles were used to display the quantity in each treatment group. The quantity of OsHV-1 DNA at the time of death was compared using a general linear model with the factors OsHV-1 isolate, injection dose, and challenge method, with tank as a random effect. Differences in the quantity of viral DNA between groups were assessed using the least significant difference method. 

## 3. Results

In vivo amplification of OsHV-1 was achieved from the three archived samples of whole oyster tissues. There was 50% mortality and 100% morbidity (gaping) 72 h after injection of primary inoculae for each sample. The average quantity of OsHV-1 DNA in gill and mantle tissue was >10^4^ genome copies.mg^−1^ (Table 1).

**Figure 2:**
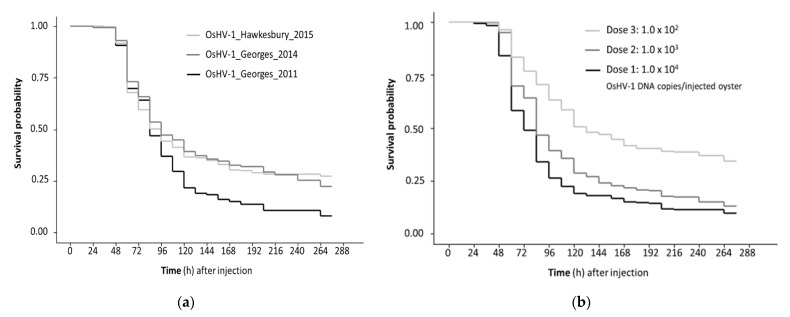

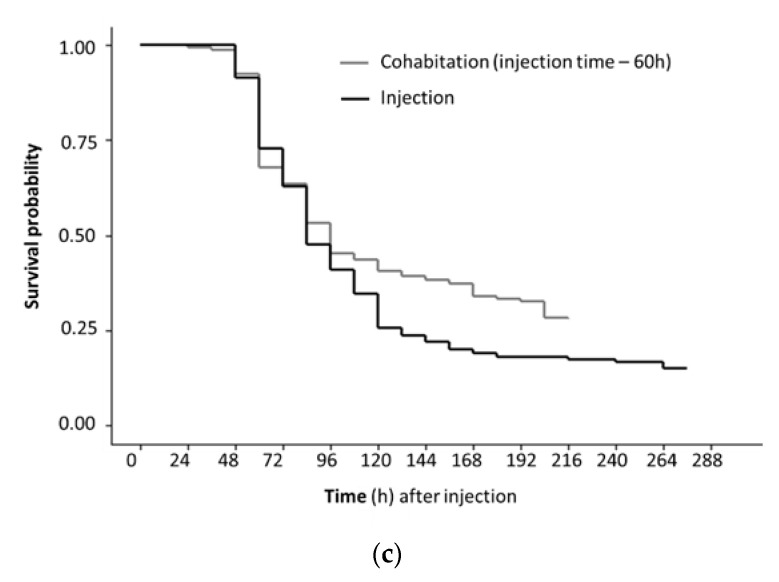
Kaplan Meyer survival curves for three OsHV-1 isolates after Pacific oysters were challenged by injection and cohabitation. Survivor probability was determined using secondary inoculae prepared as tissue homogenates from Pacific oyster challenged by injection with archived field samples. (**a**) Comparison of OsHV-1 isolates collected at different times from two different rivers; (**b**) Comparison of challenge method with time adjusted by −60 h for cohabitating oysters to allow for the longer incubation period with exposure from injected oysters; (**c**) Comparison of different doses used for the half of the oysters in each replicate tank that were challenged by injection. Data from replicate tanks were pooled. Each factor was significant (log rank test *p* < 0.05).

When oysters were challenged with the secondary inoculae, mortality was first observed in injected oysters 48 h post-injection (pi) and 36 h after that in the cohabitating oysters. Mortality peaked between 48 and 144 h pi, with low level mortality continuing until the completion of the trial at 276 h pi (Figure 2). The total cumulative mortality was significantly higher for the 2011 isolate (OsHV-1_Georges_2011; 90.3%) compared to the 2014 (OsHV-1_Georges_2014; 74.3%) and 2015 isolates (OsHV-1_Hawkesbury_2015; 74.8%) (Table 2). Although, there was variation between the replicate tanks and the different starting doses (Table 2). Considering the range of doses and the challenge method, the survivor probability was lower for the isolate OsHV-1_Georges_2011 compared to OsHV-1_Hawkesbury_2015 (Table 3).

The mortality of oysters challenged by injection was higher than by cohabitation, irrespective of isolate and dose (Figure 2a). Mortality was dose responsive with the lowest dose used (10^2^ OsHV-1 DNA copies per injected oyster) close to the 50% lethal dose in this experimental setting (Figure 2b). Oysters injected with the lowest dose had a higher survivor probability compared to oysters injected with the higher doses of OsHV-1. The survivor probability was also higher for oysters challenged by cohabitation compared to injected oysters (Figure 2c).

The hazard of death for oysters challenged with the 2011 isolate (OsHV-1_Georges_2011) was significantly higher than for the 2015 isolate (OsHV-1_Hawkesbury_2015) (Table 3). The two Georges River isolates (2011 and 2014) were not discernibly different in this standard infection model. This was influenced by the lowest injection dose for the 2014 isolate not generating any mortality in one of the replicate tanks. However, there was evidence of infection in this tank with OsHV-1 replication observed in the injected oysters (3/5 tested positive; up to 4.0 × 10^4^ OsHV-1 DNA copies/mg). There was also transmission to the cohabitating oysters with 2/5 testing positive (1.6 × 10^3^ and 5.8 × 10^3^ OsHV-1 DNA copies/mg). 

**Figure 3:**
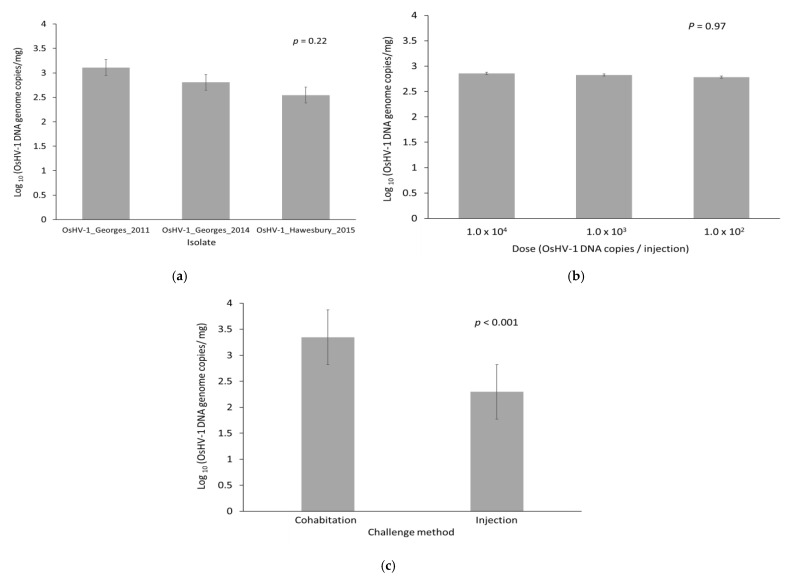
Estimated quantity of OsHV-1 DNA at the time of death in oysters that died following challenge by injection or cohabitation. (**a**) OsHV-1 isolate; (**b**) Dose of OsHV-1 for oysters that were challenged by injection; (**c**) Challenge method. Data are the predicted mean and standard error from a general linear model.

The quantity of OsHV-1 DNA in oysters that died was 1.4 × 10^3^–3.8 × 10^3^ OsHV-1 DNA copies/mg (95% confidence interval for the mean) at the time of death. This was much higher compared to the quantity present in oysters that survived to the end of the trial and were positive for OsHV-1 (2.5 × 10^1^–1.0 × 10^2^ OsHV-1 DNA copies/mg, *p* < 0.001). The prevalence of OsHV-1 in surviving oysters at the end of the trial was low (12%). There was no difference in the quantity of OsHV-1 DNA at time of death for the three different isolates (Figure 3a; *p* = 0.22). For those oysters that died, the initial injection dose did not impact the amount of viral DNA at the time of mortality (Figure 3b; *p* = 0.97). However, oysters challenged by cohabitation had a higher viral load (3.36 × 10^3^) compared to those challenged by injection (2.30 × 10^2^) (Figure 3c; *p* < 0.001). The cause of death of all challenged oysters in this trial was attributed to OsHV-1.

There was no mortality in oysters challenged by injection with the negative control or the oysters cohabitating with these, and all qPCR tests for controls were negative for OsHV-1.

## 4. Discussion

This study demonstrated different disease phenotypes for OsHV-1 isolates using a standardized laboratory infection model to compare peak viral load, transmissibility, and oyster survival. An increased hazard of death was shown to be associated with an isolate obtained shortly after the index case of POMS in Australia (OsHV-1_Georges_2011) compared to a later OsHV-1 isolate from the Hawkesbury River (OsHV-1_Hawkesbury_2015). This method will be useful for further assessment of the phenotypic relevance of the considerable genotypic diversity within OsHV-1 [11,30,31]. Variation in the complete genomes of OsHV-1 indicate several factors which are likely to impact virulence, including the presence/absence of some genes, variability in the amino acid sequence coded by open reading frames, and variability in regions that function to regulate viral gene expression [32]. The genotypic variation in OsHV-1 provides a mechanism for viral evolution which can be influenced by host species as well as temporal and geographic separation [42]. Considerable selection pressure was expected for the OsHV-1 microvariant in Australia, through seasonal recurrence with a substantial reduction in the size of the Pacific oyster population in the NSW estuaries where it has become endemic [16]. The phenotypic differences between the isolates of OsHV-1 in this trial give rise to several research questions about the genomic mechanisms and options for industrial applications of low or avirulent field strains in disease management.

Considerations of the virulence of OsHV-1 should embrace an understanding of the polymicrobial nature of the pathogenesis of the disease. It has been reported that OsHV-1 causes disease through immune suppression leading to a fatal bacteremia [17]. Interestingly, the difference in pathogenicity of the OsHV-1 isolates in the present study did not correspond to differences between the peak viral load in oysters that died. A low dose of OsHV-1 (10^2^ viral copies per injection) resulted in a lower hazard of death than higher doses (10^3^–10^4^ viral copies per injection), consistent with previous trials [43]. Although there was a clear dose-dependent response, the initial injection dose did not impact the amplification of OsHV-1 to peak titre. Furthermore, there was more OsHV-1 DNA in oysters that died after exposure by cohabitation compared to those which were injected, despite the injected treatments having a higher hazard of death. These key findings support an understanding that mortality resulting from OsHV-1 infection is more complex than the amount of virus that is amplified [44]. For example, previous studies indicate that environmental factors can alter the composition of the microbiome associated with oysters which in turn influences the course of disease [45] and, currently, there are no histopathological lesions that are specific for OsHV-1 disease [46]. The influence of energetic reserves on metabolism [14] and immune mechanisms, such as autophagy [47], are also key to the expression of disease. The present study design was able to control for these factors by keeping host and environment constant.

Increased virulence of microvariant OsHV-1 led to these genotypes dominating samples collected from diseased oysters in France after 2008, largely replacing the reference genotype [4,11]. The genotypic changes contributing to higher virulence included a deletion in the microsatellite locus upstream and several polymorphisms in ORF4 [11] but greater complexity of OsHV-1 genotypic variation was documented around this time [48]. The interaction and competition between all genotypes in the field remains unknown. Evidence of co-infection with a microvariant and reference genotype in subclinical infection [49] suggests that multiple strains can cause infection during a single season. Importantly, there was higher genotypic diversity in OsHV-1 from apparently healthy oysters in Italy compared to viruses characterized elsewhere from disease outbreaks [31]. It was hypothesized that the evolution of OsHV-1 into a more virulent genotype may have developed in Europe due to the intensive farming resulting in an increased number of susceptible hosts and increased animal movement [32]. In some cases, conditions suitable for OsHV-1 with high virulence are encouraged by the practice of stocking greater numbers of oysters on farms to compensate for disease losses [3]. Conversely, Pacific oyster farming in the OsHV-1 affected Georges and Hawkesbury River estuaries in Australia was replaced by the non-susceptible oyster species, *Saccostrea glomerata*, resulting in a dramatically reduced potential host population. Here, a declining trend in cumulative mortality and prevalence of OsHV-1 [16] might reflect a shift towards an attenuated or avirulent genotype more suited than the highly virulent microvariant isolates for the changed conditions [16]. Further, there is evidence that the recurrent seasonal outbreaks reported in Europe and Australia are perpetuated by subclinical OsHV-1 infection during cooler months [49,50], favoring long-term success of OsHV-1 with lower virulence. 

Several years elapsed between the collection of the microvariant OsHV-1 isolates examined in this study. This provided considerable opportunity for evolution for a virus that can replicate to high peak titre less than two days after infecting a host [44]. Even though the present study was biased towards collection of OsHV-1 from clinical disease, lesser virulence corresponded with the later timepoint. Viral attenuation might be attributed to the “trade-off hypothesis”, where benefits to the fitness of a virus are obtained through reduced virulence at the expense of reduced transmissibility [51]. Whole genome and viral transcriptome sequencing are recommended to identify genotypic differences that can be tested to explain differences in the phenotype of OsHV-1. This knowledge, and a better understanding of the field ecology of OsHV-1, will be important for disease management where OsHV-1 is endemic because eradication of OsHV-1 is impractical due to expense and the high risk of re-infection [52]. Characterization of attenuated OsHV-1 strains by genome analysis and phenotyping studies may lead to methods for disease management in commercial farms through controlled exposure. Preliminary studies using virulent OsHV-1 under a specific temperature regime has shown that this is possible [53].

## 5. Conclusions

Variation in the virulence of isolates of OsHV-1 was identified in this study that matched epidemiological observations of waning disease severity over time in the sampling location. This was despite using isolates obtained in surveys biased towards detection of pathogenic types of OsHV-1. Considerable genetic diversity of OsHV-1 is being documented globally together with selection pressure for viral fitness associated with changes to management of Pacific oyster farms. While genotyping was beyond the scope of this study, genotypic comparison of isolates of varying virulence may lead to identification of the virulence factors in OsHV-1. This may lead to new management strategies which utilize controlled exposure to attenuated strains of OsHV-1.

## Figures and Tables

**Table 1:** Archived oyster tissue samples (−80 °C) collected in the field from which primary inoculae (clarified and filtered mantle and gill homogenates) were prepared for injection of naïve Pacific oysters (*n* = 8 per group). These experimentally infected oysters provided the first in vivo passage OsHV-1 (secondary inoculae) used to compare the isolates.

Region Sampled	Date Collected	Clinical Signs (%) ^1^	OsHV-1 qPCR Positive (%)	OsHV-1 Quantity ^2^	Identity in Further Evaluation
Georges River, NSW	24/11/2011	100	100	1.15 × 10^4^	OsHV-1_Georges_2011
Georges River, NSW	24/02/2014	100	100	4.01 × 10^4^	OsHV-1_Georges_2014
Hawkesbury River, NSW	11/11/2015	100	100	4.76 × 10^4^	OsHV-1_Hawkesbury_2015

^1^ The experiment was completed when mortality was 50% when the remaining oysters were examined for clinical signs (gaping); ^2^ Average OsHV-1 genome copies/mg in gill and mantle tissue.

**Table 2:** Total cumulative mortality in duplicate tanks for each isolate with three different doses for injection. Approximately half of the oysters in each tank were challenged by injection and the remainder were challenged by cohabitation in the same tank.

Isolate	Dose ^1^	Total Cumulative Mortality (%)				
		Range in Duplicate Tanks (%)	Injected Oysters		Cohabitating Oysters	
			n	Mortality (%)	n	Mortality (%)
OsHV-1_Georges_2011	1.0 × 10^4^	90–93	20	90	19	89
			21	100	19	84
	1.0 × 10^3^	92–95	19	95	19	89
			24	100	15	87
	1.0 × 10^2^	83–90	22	91	18	72
			21	95	18	83
OsHV-1_Georges_2014	1.0 × 10^4^	79–93	20	85	19	74
			22	95	18	89
	1.0 × 10^3^	87–88	20	100	23	78
			20	90	19	84
	1.0 × 10^2^	0–84	19	89	18	78
			19	0	20	0
OsHV-1_Hawkesbury_2015	1.0 × 10^4^	88–90	23	91	18	83
			25	88	16	94
	1.0 × 10^3^	69–75	20	90	16	56
			19	100	20	40
	1.0 × 10^2^	46–78	23	61	18	100
			20	60	21	33

^1^ OsHV-1 DNA copies/injected oyster.

**Table 3:** Hazard ratios predicted from a Cox proportional hazards model.

Factor	Level	Hazard Ratio	
		Point Estimate	95% Confidence Interval
Isolate	OsHV-1_Georges_2011	1.37	1.01–1.86
	OsHV-1_Georges_2014	1.06	0.63–1.79
	OsHV-1_Hawkesbury_2015 ^1^	-	-
Challenge method	Injection	1.30	1.02–1.65
	Cohabitation ^1^	-	-
Dose (OsHV-1 genome copies/injected oyster)	1.0 × 10^2^	0.44	0.24–0.79
	1.0 × 10^3^	0.78	0.57–1.07
	1.0 × 10^4 1^	-	-

^1^ Reference category.

## Data Availability

The data presented in this study are available on request from the corresponding author.
